# Algorithmic profiling as a source of hermeneutical injustice

**DOI:** 10.1007/s11098-023-02095-2

**Published:** 2024-02-05

**Authors:** Silvia Milano, Carina Prunkl

**Affiliations:** 1 University of Exeter, Exeter, UK; 2 Utrecht University, Utrecht, Netherlands

**Keywords:** Epistemic injustice, Hermeneutical injustice, Algorithmic profiling; Epistemic fragmentation, Ethics of AI

## Abstract

It is well-established that algorithms can be instruments of injustice. It is less frequently discussed, however, how current modes of AI deployment often make the very discovery of injustice difficult, if not impossible. In this article, we focus on the effects of algorithmic profiling on epistemic agency. We show how algorithmic profiling can give rise to epistemic injustice through the depletion of epistemic resources that are needed to interpret and evaluate certain experiences. By doing so, we not only demonstrate how the philosophical conceptual framework of epistemic injustice can help pinpoint potential, systematic harms from algorithmic profiling, but we also identify a novel source of hermeneutical injustice that to date has received little attention in the relevant literature, what we call epistemic fragmentation. As we detail in this paper, epistemic fragmentation is a structural characteristic of algorithmically-mediated environments that isolate individuals, making it more difficult to develop, uptake and apply new epistemic resources, thus making it more difficult to identify and conceptualise emerging harms in these environments. We thus trace the occurrence of hermeneutical injustice back to the fragmentation of the epistemic experiences of individuals, who are left more vulnerable by the inability to share, compare and learn from shared experiences.

## Introduction

It is well-established that algorithms can be instruments of injustice. It is less frequently discussed, however, how current modes of AI deployment often make the very discovery of injustice difficult, if not impossible. In this article, we focus on the effects of algorithmic profiling on epistemic agency. We show how algorithmic profiling can give rise to *epistemic injustice* through the depletion of epistemic resources that are needed to interpret and evaluate certain experiences. By doing so, we not only demonstrate how the philosophical conceptual framework of epistemic injustice can help pinpoint potential, systematic harms from algorithmic profiling, but we also identify a novel *source* of hermeneutical injustice that to date has received little attention in the relevant literature: *epistemic fragmentation*. As we explain below, epistemic fragmentation is a structural characteristic of algorithmically-mediated environments that isolate individuals, making it more difficult to develop, uptake and apply new epistemic resources. This, in turn, can impede the identification and conceptualisation of emerging harms in these environments. We trace the occurrence of hermeneutical injustice back to the fragmentation of the epistemic experiences of individuals, who are left more vulnerable by the inability to share, compare, and learn from shared experiences.

To recognise injustice, we fundamentally rely on our epistemic practices and institutions. Sometimes, however, it is the epistemic practices themselves that contribute to the perpetuation of injustice. *Epistemic injustice* was introduced by Miranda Fricker (2007) to describe a “distinctly epistemic kind of injustice” (Fricker, 2007, p.1). It involves an injustice done to someone *in their capacity as a knower* or as a *transmitter of knowledge*. While Fricker was the first to use the term ‘epistemic injustice’, the phenomenon itself has been discussed much earlier by feminists of colour, as pointed out by McKinnon (2016, p.438). These include Moraga and Anzaldúa (1981), Hooks (1992), Alcoff (1996), and Collins (2000). In the following, we examine whether and how algorithmic profiling—the automated process of extrapolating information about a person on the basis of personal data—gives rise to epistemic injustice. We discuss several ways in which epistemic agency is undermined and show how algorithmic profiling can prevent individuals from accessing and meaningfully interpreting important aspects of their experiences (Fricker, 2007; Medina, 2017; Pohlhaus, 2017).

As one of the driving factors for epistemic injustice in this context, we highlight the so-called *epistemic fragmentation* of the human subjects of algorithmic profiling (Milano et al., 2021): as personalisation progresses, both online and in the interaction with algorithmic systems that are capable of producing individualised predictions (for example in healthcare or insurance applications), the experience of individuals interacting with algorithmic systems has increasingly become unique and individualised. We will discuss in detail, how such individualisation hinders people and communities from meaningfully sharing and comparing their experiences. Sharing and comparing form integral parts of our epistemic practices and institutions. In this context, we point to the importance of an epistemic infrastructure that enables such sharing and comparing. We identify a lack of such epistemic infrastructure as a source of epistemic injustice. This kind of structural dysfunction can give rise to epistemic injustice, we argue, because it (i) impairs the exercise of our distinctly epistemic capacity for enquiry; and (ii) via this impairment constitutes a harm that disproportionately affects marginalised groups.

Algorithmic systems have far reaching effects and can be implicated in epistemic injustice. This has also been discussed by various authors, who highlight relationships between new digital technologies and cases of epistemic injustice. Origgi and Ciranna (2017) discuss the risk of our online personas creating a “representational crisis” (p.309) in users if there is a dissonance between these online personas and how individuals perceive themselves and their experiences offline. They argue that this creates a double case of hermeneutical injustice (a) through a loss of hermeneutical control of individuals over their online identities, and (b) through the lack of hermeneutical resources that would allow individuals to discuss epistemic implications of digital realities. While we equally emphasise (b) in this article, our focus of attention differs from that of Origgi and Ciranna (2017), who, similarly to Scotto (2020), are mainly concerned with alienation and epistemic confidence in self-knowledge. Instead, we are interested in how (b) comes about and how it affects the experience of individuals more generally. Symons and Alvaro (2022) link the emergence of epistemic injustice—both hermeneutical and testimonial—to algorithmic opacity. While opacity plays an important role (see Sect. 5), we instead focus on the relationships *between* individuals and the importance of communication infrastructure. Finally, Oliphant (2021) links epistemic injustice to information landscapes, emphasising how conceptualising individuals as “users” of algorithmic systems erodes their epistemic agency.

We begin this article with an introduction to the concept of *epistemic injustice*. Our focus here will be on *hermeneutical* injustice—a form of epistemic injustice that arises if a lacuna in the collective epistemic resources systematically prevents individuals from making sense of their social experiences (Fricker, 2007; Medina, 2017; Pohlhaus, 2017). Section 3 proceeds with an introduction to algorithmic profiling and discusses various ways in which it can affect epistemic agency. Section 4 introduces the concept of *epistemic fragmentation*. In Sect. 5, we argue that algorithmic profiling can give rise to hermeneutical injustice. We demonstrate how individuals subject to profiling and online personalisation routinely lack access to the epistemic resources needed to interpret their experiences and show how this leads to epistemic injustice. Finally, we discuss epistemic fragmentation as one of the driving factors of epistemic injustice in the online context and highlight the importance of structural conditions and provide an outlook for further equiry in Section 6.

## Hermeneutical injustice

*Epistemic injustice* describes a form of injustice that is distinctly epistemic in that it involves a wrong done to someone in their capacity as an epistemic agent (Fricker, 2007). It relates to practices of communication, meaning-making, and knowledge production and addresses the unwarranted exclusion of individuals from these practices (Kidd et al., 2017). Fricker distinguishes between *testimonial* and *hermeneutical injustice*. Testimonial injustice describes the discrediting of a speaker’s words as a result of identity prejudice, *e.g.* as relating to ethnicity, gender, or religion. Through such devaluation, the speaker is prevented from contributing to the epistemic system (Dotson, 2014), *i.e.* transmitting their knowledge to others via testimony, and excluded from existing epistemic practices.

Hermeneutical injustice, on the other hand, involves a “structural prejudice in the economy of collective hermeneutical resources” (Fricker, 2007, p.1) that results in groups or individuals being prevented from making sense of significant aspects of their experience through a lack of collective conceptual or interpretative resources. In this case, there is a lacuna in the epistemic system that prevents them from meaningfully interpreting their experiences. Fricker gives as an example the historical case of Carmita Wood, a woman who had been subject to what we would now refer to as sexual harassment, but which at the time was not a named or identified phenomenon. Carmita was unable to both interpret and express her experience of sexual harassment because of a gap in the collective hermeneutical resources prevented her from accessing the very concept of sexual harassment. Importantly, such collective hermeneutical resources are *socially produced*—the epistemic efforts required for their production are fundamentally collective. Structural prejudices that are present in society affect which epistemic resources are available. The case of sexual harassment exemplifies this: women as a category have been historically subjected to oppression and prejudice, and the lacuna is a manifestation of an imbalanced, sexist power structure. In the following, we show how algorithmic profiling may prevent individuals from making sense of their experiences in a similar way to what Carmita Wood was experiencing, but we argue that, in our case, what *causes* the hermeneutical injustice is importantly different.

Medina (2017) identifies two varieties of hermeneutical injustice: *semantic* and *performatively* produced hermeneutical injustice. Performatively produced hermeneutical injustice occurs when individuals are judged as unintelligible because of their “communicative performance or expressive style” (Medina, 2017, p.46). For example, it has been argued that women have a different expressive style when it comes to ethical judgments, which is sometimes marginalised as morally immature (Fricker, 2007, p.160), thereby excluding them from contributing to the hermeneutical system. Semantically produced hermeneutical injustice, on the other hand, occurs when there is a gap in the collective conceptual vocabulary, an “unavailability of labels” (Medina, 2017, p.45). The example relating to sexual harassment given above described such a semantically produced injustice: Carmita Wood was unable to articulate her experience because the conceptual vocabulary referring to sexual harassment simply did not exist. Semantically produced epistemic injustice is the result of a dysfunction in the *formation* of epistemic resources, such as the concept of sexual harassment. There can also be a dysfunction in the *uptake* of conceptual vocabulary into the collective epistemic resources, for example through what Medina (2017) has called *hermeneutical marginalisation*. Here, certain individuals and groups are systematically prevented from contributing to the production of shared epistemic resources. In these circumstances, the affected communities are not supported in their contribution of relevant epistemic resources. As will be shown below, there can also be a dysfunction in the *application* of available concepts. Here, instances of injustice similarly result from a lacuna in the hermeneutical resources. Yet, this lacuna is not necessarily the result of a gap in the collective conceptual vocabulary. Instead, it results from an inability to correctly apply a concept in the right context.

Epistemic infrastructure is a necessary requirement for the exchange of knowledge. It can take on different forms: access to epistemic institutions, such as schools, universities, or libraries may be considered as forming as much part of this infrastructure as social networks. In the case of Carmita Wood, it was only after Wood had the opportunity to speak to Lin Farley, then director of Cornell’s *Women’s Section*, that she was able to understand her experience of sexual harassment. Farley, on the other hand, had had discussions about unwanted sexual advances with the students in her seminar. These discussions allowed her and her students to evaluate their collective experiences, eventually leading to their coining the term ‘sexual harassment’. Importantly, this process relied on the existence of a forum for communication that enabled participants to share and compare their experiences. Through her seminar, Farley had created such a forum. The infrastructure in this case was provided through the university and the outreach activities of Farley herself. Epistemic infrastructure, however, may equally be entirely virtual, as powerfully demonstrated by the effects of the internet and its online social media platforms. Never has it been easier for individuals (with internet access) who are part of a marginalised group to make contact with peers and to freely share their experiences, thereby constantly enriching the pool of hermeneutical resources.

**Fig. 1:**
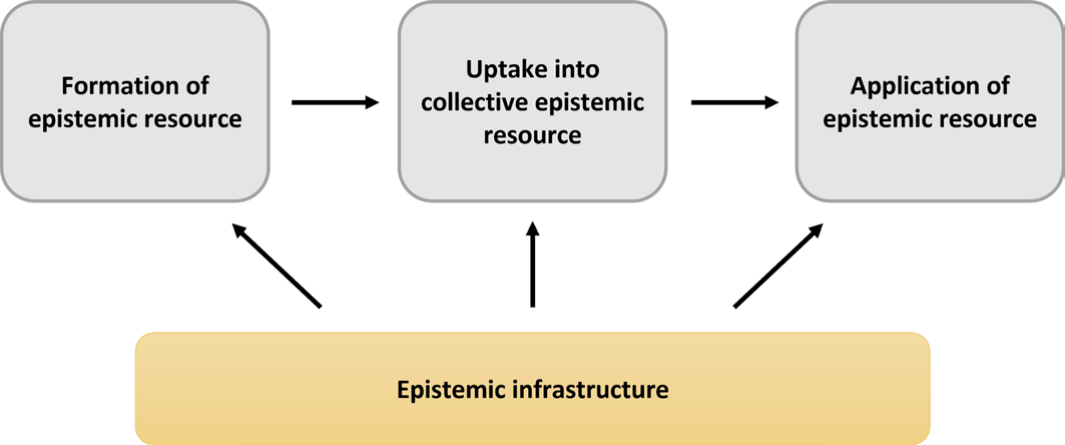
The diagram illustrates the different steps involved in the formation, collective uptaking, and application of epistemic resources. Sources of epistemic injustice are linked to dysfunctions within the various steps. Underlying the entire process is the epistemic infrastructure that creates opportunity for the sharing and comparing of experiences, thereby enabling epistemic resources to be created, taken up, and applied

In Fig. 1, we illustrate the fundamental role of epistemic infrastructure for the formation of epistemic resources. We deliberately chose a heuristic that outlines the different steps relating to the formation and availability of collective hermeneutical resources. Naturally, our depiction of epistemic resource creation is highly idealised and should be regarded as such. In reality, the formation of shared epistemic or cultural resources is a much more complex and messy process.

### Formation

At the beginning of the process there is the formation of a given epistemic resource, *i.e.* the identification of the concept. This step sometimes involves giving a name, for instance the coining of the term ‘sexual harassment’. Semantically produced epistemic injustice, as outlined above, relates to a dysfunction in this process which in turn leads to hermeneutical disadvantages and harms. It points to instances where the conceptual vocabulary either is unavailable or is available but excludes things that really ought to fall under its umbrella (Medina, 2017, p.45).

### Uptake

The second step in the diagram describes the recognition of the concept as part of the collective hermeneutical resources. It illustrates that certain conceptual resources might very well be part of the hermeneutical resources available to members of a particular group, but not to the collective (Dotson, 2014; Medina, 2017; Pohlhaus, 2012). There are different possible causes for such lack of collective uptaking of hermeneutical resources. For example, the dominantly situated group might refuse to acknowledge epistemic tools developed by those situated marginally (Medina, 2017; Pohlhaus, 2012). This includes cases where those situated marginally are misheard or judged unintelligible due to their expressive style (Medina, 2017). In both cases, epistemic resources of group members are marginalised and not taken up by the dominant group.

### Application

The third step in the diagram relates to the actual application and internalisation of epistemic resources. Here, too, there can be dysfunctions. In the Netfilx series ‘Sex Education’, a character named Aimee is sexually assaulted in the bus. She is traumatised by the experience but does not know how to make sense of it, including how to talk about it. In this case, the concept itself, *i.e.*, ‘sexual abuse’, is readily available and moreover exists as part of the shared hermeneutical resources in this setting. Yet, Aimee is unable to successfully apply the concept to her *own* experiences. Research shows that this kind of psychological response is common among victims (see Gardiner, 2021, and references therein), possibly as a result of the cognitive dissonance engendered by the necessity to preserve one’s sense of integrity, on the one hand, and the recognition that this has been compromised by the assault.

### Epistemic infrastructure

Finally, underlying the entire process is the existence and functionality of epistemic infrastructure, including what Crerar calls an *expressively free environment* in which “a particular experience that individuals or groups have a significant interest in coming to understand can be discussed in hermeneutically conducive ways” (Crerar, 2016, p. 205). It is this infrastructure that enables and supports the formation of hermeneutical resources, the uptake of these resources into the collective hermeneutical repertoire, and their application. We notice that dysfunctions of the epistemic infrastructure can underpin the other three identified sources of hermeneutical injustice.

## Epistemological concerns with algorithmic profiling

We now introduce the practice of algorithmic profiling and point out different ways in which it can have negative impacts on epistemic agency, *i.e.* our capacity for enquiry (in addition to other well-known and documented instances of harm, for example through bias). Algorithmic profiling refers to the automated process of extrapolating information about a person based on personal data. We mainly focus on profiling by machine learning (ML) systems. These systems use input (training) data to build predictive models, which in turn allows them to make statistical inferences (predictions) when confronted with new data. Algorithmic profiling is used by numerous services to predict, among other things, preferences, medical needs, performance, or interests. Such predictions allow for the optimisation and personalisation of various services. In this article, we will limit our attention to *online personalisation*, which is widespread across social media platforms, search engines, online stores, or news pages. In these cases, algorithmic profiling is used to determine which posts, search results, products, or news a given user sees at a given time.

From an epistemological perspective, there are (at least) two distinct sets of risks associated with algorithmic profiling: *inference* and *enquiry*. The first set, *inference*, points to the various shortcomings related to the extrapolation of information from collected datasets that might result in flawed, wrong, or inadequate profiles. For example, it has been shown that setting the gender to ‘female’ on Google’s AdSetting’s page results in fewer advertisements for high-paying jobs (Datta et al., 2015). One potential reason for such shortcomings is low data quality. Data might be corrupted, incomplete, biased, or simply inadequate for the task. Another potential reason is the wrong choice of proxies for a given feature. In 2019, for example, it became public that a US health-care algorithm systematically assigned a lower health risk to people who identified as black than to equally ill people who identified as white (Ledford, 2019). In this case, one of the proxies for health was total health-care cost accrued in one year. The system did not take into account, however, that accessibility to healthcare varies within the population and that, as a result, people who identified as black did not make use to the same extent of health-care facilities as white people. In this case, the inference drawn about the health status of people was inaccurate. These and other types of inference-level problems are extensively researched in the fairness literature, in particular the literature on non-discrimination law and computer science (see e.g. Barocas & Selbst, 2016; Wachter, 2020). It is generally recognised that purely technical solutions cannot be given (Le Bui & Noble, 2020; Wachter, 2021). One issue in particular arises from the statistical nature of ML reasoning. If ML driven algorithms are considered as epistemic resources that help us to improve understanding and/or make better predictions, then the statistical nature of ML de facto prevents groups underrepresented in the data from contributing to this epistemic resource on an equal ground to dominant groups (Gebru et al., 2021), simply because they are not equally captured in the data sets used to train the predictive algorithms, or because the quality of the data pertaining to them is not adequate, due to biased data gathering or reuse practices (Leonelli et al., 2021). This effectively can be regarded as an instance of unjust hermeneutical marginalisation, in that it constitutes a situation of unequal hermeneutical participation (Fricker, 2016, p.163).

The second set of epistemological concerns relates to the way in which algorithmic profiling affects us as epistemic agents through influencing our capacity for reasoning and understanding. We may call this the level of *enquiry*. While, as already mentioned above, the internet and social media have opened up many opportunities that are beneficial to enquiry, we now outline some of the mechanisms that can undermine epistemic agency.

Algorithmically generated profiles and profiling categories are often not epistemically accessible to individuals subject to profiling. This might be the case for several reasons: (a) individuals are not aware that they are subject to profiling; (b) they are aware that they are subject to profiling but cannot access their profiles due to technical or institutional hurdles; (c) they can access their profiles but cannot meaningfully interpret their profiles or the inferences that are performed on their basis. In the case of the US health-care algorithm described above, patients weren’t aware that they were subject to algorithmic decision-making (a). However, even if they *had* been aware, they might not have been granted access to their profiles (b). In some cases, the complex nature of the algorithm and the way it weighs large sets of different variables, make it impossible for humans, let alone laypersons, to understand why a certain algorithmic decision was made (c) (Selbst & Barocas, 2018).

A further issue that arises at the level of enquiry is not so much about epistemic access to the algorithmic decision-making itself (though this may certainly contribute), but rather about the ability to meaningfully interpret one’s experiences in light of the individualised nature of these experiences. It is to this issue that we now turn our focus.

## Epistemic fragmentation

An important consequence of the increasing personalisation of online content through algorithmic profiling is *epistemic fragmentation* (Milano et al., 2021). We take epistemic fragmentation to be a state in which individual epistemic agents have no (or severely limited) access to information about other individuals’ personal contexts. A personal context, in turn, is the sum of an individual’s personal information (for example, information about one’s identity, beliefs, interests, and personal characteristics) and the information about other entities that the same individual is exposed to in a given context. For example, my personal context as I sit in a seminar room includes information about myself (my identity, personal characteristics, present state of mind, etc.) in addition to my surroundings (*e.g.*, the content of the seminar presentation, other participants, etc.). In this case, the seminar participants have a lot of shared context: among other things, each one is aware of the presence of the others, and the content of the presentation is public to everyone. As a result, the state of the seminar room participants is not epistemically fragmented. Contrast this case with online browsing on YouTube: there, an individual viewer who accesses a video through the platform’s inbuilt recommender consumes the content in isolation. While the same content may be recommended to others, each viewer does not have access to aspects of others’ personal context.

With epistemic fragmentation, individuals who are subject to profiling and unfairly impacted by it, undergo these experiences in isolation and lack the epistemic means (*e.g.*, awareness) necessary to share their experiences within their social circles. For example, if a person is exclusively shown advertising for low-paying jobs, their epistemic resources may not allow them to meaningfully interpret their online experience: is everyone being shown the same jobs? Why are they all low paying? These questions might arise if the individual stops to reflect critically on what they experience, which is rarely the case. More often than not, the individual just accepts (or has to accept) their experience, without further interrogation. In the case of the flawed health-care algorithm discussed above, it was not the patients themselves who raised the alarm about the unfair treatment. Instead, it was a group of scientists running routine statistical checks on healthcare data provided by a large hospital, who came across the disparities. In the case of the discriminatory Google Ads, it was equally a team of scientists who analysed the relationship between online advertisement and gender. The conceptual resources individuals need to make sense of their experiences are developed through shared social interactions. Epistemic fragmentation undermines the abilities of individuals to develop and access an understanding of their experiences, as well as to help others understand and validate their own experiences.

Epistemic fragmentation can be an indication of a shortcoming in communication infrastructure. In turn, it can lead to semantically produced epistemic injustice by preventing the meaningful exchange of experiences that would enable the creation of adequate conceptual resources. In a similar fashion, it can also prevent the uptake of epistemic resources in the sense that, even if conceptual resources are readily available, their collective uptake might be hindered. In the case of low-wage job advertisement, the conceptual resources to identify this as a case of gender-based discrimination exist and are in principle available. However, since individuals are isolated with their experience and usually unaware of what is happening, they do not participate in activities of sharing and comparing. This, in turn, is a factor preventing them from meaningfully placing their experience within the wider hermeneutical context. Finally, epistemic fragmentation may also contribute to undermining the application of epistemic resources. Users may well be aware that, say, discrimination on the basis of race is an issue that frequently occurs within the context of algorithmic profiling. Yet, they may not be aware that it applies to *them*. In the Netflix series, Aimee eventually comes to terms with the fact that she was a victim of sexual assault because her friends noticed behavioural changes and quizzed her about her experiences. In an online context, as we argue next, there can be obstacles to this process of recognising and applying concepts to one’s online experience.

In the next section, we will show how this can amplify existing patterns of discrimination (by making it harder for individuals to gain awareness of their own social circumstances), as well as creating the conditions for the emergence of new forms of unfair targeting, for example on the basis of inferred interests or behavioural vulnerabilities. These harms are difficult to identify, without access to individuals’ experiences, and recourse may be especially difficult to come by, since it is difficult to prove when they happen and the extent to which individuals may be harmed.

## Algorithmic epistemic injustice

In the previous section, we reviewed epistemological concerns with algorithmic profiling and sketched how epistemic fragmentation impacts an epistemic agent’s means of enquiry. In this section, we turn to examine how this can create, or help maintain, epistemic injustice by damaging the epistemic infrastructure in ways that can impair the formation, uptake, and application of new epistemic resources.

### From fragmentation to injustice

Epistemic fragmentation can sometimes be a welcome feature of an epistemic environment: for example, the anonymity it affords can allow space to explore new ideas or develop one’s opinion on a topic without fear of undue repercussions. However, as we have argued in the previous section, it can also impair our capacity for enquiry. Sharing and comparing experiences is necessary in order to identify and label common patterns, thereby creating new epistemic resources that can be used to better understand one’s experiences. The effects of epistemic fragmentation can wrong individuals who lack access to such epistemic resources which would allow them to understand harmful experiences. Over time, a lack of epistemic infrastructure, such as in the form of shared spaces where individuals can come together and co-create epistemic resources, can give rise to epistemic lacunas. Since we live in a non-ideal society whose institutions and epistemic practices have been shaped by historical injustices, we should expect this to be a source of hermeneutical injustice. In the aforementioned case of sexual harassment, for instance, the ability to access women’s groups and openly share experiences was instrumental to identifying an otherwise overlooked and under conceptualised pattern of sexual harassment. But in epistemically fragmented contexts, coming together in this way could be more challenging, since individuals lack a shared context.

### Epistemic fragmentation and epistemic lacunas

Fricker’s discussion of the identification of the concept of sexual harassment illustrates one way in which epistemic fragmentation can sustain hermeneutical injustice, by blocking avenues to fill the epistemic lacunas that are necessary to make sense of individual experiences. In this way, epistemic fragmentation can contribute to maintaining what Medina calls *semantically produced* hermeneutical injustice. As we have seen above, this type of injustice occurs when a useful concept is missing from the collective repertoire, just as the concept of sexual harassment was unavailable before its introduction by the women’s movement. Identifying clear examples of semantically produced hermeneutical injustice is often only possible in retrospect, since identifying a lacuna already requires *some* awareness that epistemic resources are missing.

When we interact with algorithmic systems that make decisions about us based on profiling, we need to trust that this is done fairly. For example, can one be confident that the decision to reject a candidate for a bank loan didn’t hinge on protected characteristics? Can I trust that the job postings I see advertised on my personalised social media page are the best opportunities for me, or am I in fact being shown lower-paying ones because I am profiled as a woman (Datta et al., 2015).

If I don’t have meaningful access to my profile and don’t know how others are profiled, the lack of a shared context limits my ability to understand how my experiences compare to others’ and my ability to communicate their differences and similarities. Above, we discussed how algorithmic profiling can undermine epistemic agency, both through opaqueness and the resulting epistemic fragmentation. The resulting inability of individuals to draw on one’s own and others’ experiences as evidence can obscure instances of injustice that emerge as a result of algorithmic profiling. Moreover, it may obscure entirely new forms of injustice that so far have found no consideration in the general discourse. Algorithmic systems often utilise profiling categories that are far removed from attributes that most would find socially meaningful. The Facebook newsfeed algorithm, for example, profiles users according to fine-grained, machine-generated similarity categories that often are not clearly legible in terms of traditional social categories. Algorithmic systems increasingly discriminate by association, blurring the contours of the social categories that are protected (Wachter, 2020). In this context, identifying emerging cases of injustice can involve the serendipitous discovery of patterns of algorithmic decisions, which may be difficult to imagine ex ante.

This is the first way in which epistemic fragmentation produces hermeneutical injustice: by making the very *discovery* of injustice difficult. Epistemic fragmentation veils whether interactions with an algorithm are exposing someone to harm by obscuring who else might be similarly affected. In this sense, algorithmic profiling may also block the recognition of the very existence of an epistemic lacuna. This in itself is problematic, as it poses additional limits to epistemic agency. However, it becomes even more so once we consider the groups of people who are currently most affected by algorithmic discrimination. These are communities who already suffer from widespread social and structural injustice. The results of epistemic fragmentation will therefore disproportionally affect said groups, exacerbating injustice by obscuring it from scrutiny.

### Epistemic fragmentation and failures of uptake

Even when the required epistemic resources are present within society, epistemic fragmentation can put us in a position where it is difficult or impossible to adopt them, because awareness of the issues never reaches the relevant decision makers. Power relations in epistemically fragmented environments are unbalanced: the platform or service provider that uses algorithmic profiling may have access to more information about its users or targets than the individuals affected (Scotto, 2020). As Cathy O’Neil (2016) has described in a variety of settings, this imbalance often results in dismissal of the contextual evidence available to individuals:

*“An algorithm processes a slew of statistics and comes up with a probability that a certain person might be a bad hire, a risky borrower, a terrorist, or a miserable teacher. That probability is distilled into a score, which can turn someone’s life upside down. And yet when the person fights back, “suggestive” countervailing evidence simply won’t cut it. The case must be ironclad. The human victims of [harmful algorithms], we’ll see time and again, are held to a far higher standard of evidence than the algorithms themselves.”* (O’Neil 2016, p.11)

Increasingly, regulators are stepping in to require service providers to ensure equitable treatment. This tends to naturally create a top-down approach, where it is the responsibility of service providers to try to put in place measures to improve the fairness and safety of their systems. A limitation, however, comes from the fact that this setup does not afford meaningful avenues for the uptake of relevant epistemic resources that have already been identified by the affected individuals and communities.

An example where one can observe such a trend is the way social media feeds and personalised digital environments structure our individual experience of the digital world. A growing challenge to the governance of these spaces is the reliance on platform monitoring of content for the purpose of moderation. Weeding out harmful content and curating the individual experiences on the platform is an increasingly pressing issue, made more complicated by the sheer volume of content that is continuously posted online (Gorwa et al., 2020). Currently, the responsibility for moderating content posted to social media, including both content that is posted by individual users, and paid advertisement, falls mostly on the social media platforms themselves.

This moderation in turn relies heavily on automated algorithmic tools, which are used to routinely scan the vast amount of content that is posted, blocking, or flagging suspect cases that need to be referred to human moderators. The latter are then usually tasked with making final decisions about content that has been reported or flagged, including the most serious violations of content guidelines. The costs to human moderators are very high and should be acknowledged: harsh working conditions where human moderators are expected to review high volumes of extremely distressing content and make snap decisions (Gillespie, 2021). But the aspect that interests us more here is the way in which this monitoring regime impairs the uptake of new and relevant epistemic resources, leading to hermeneutical injustice.

The monitoring carried out by platforms relies on categories that are rigidly formulated and that are supposed to be applicable without a knowledge of the context in which the content is produced or consumed. This creates an environment where users of social media are treated as passive subjects, in need of protection from exposure to harmful content, instead of being empowered to contribute to the creation and modification of the existing categories used in moderation. More generally, individuals, including members of the communities that are most negatively affected by the algorithmic curation of social media, do not have the ability to contribute to the provision of epistemic resources that are used to make decisions about the content that they access.

### Hermeneutical injustice through failures of application

Epistemic fragmentation can also be a contributing factor to hermeneutical injustice through making it more difficult, or even impossible, for individuals to utilise concepts that would be necessary to make sense of their own experiences, even if those concepts are already part of the collective repertoire. We conceptualise this as a dysfunction in *applying* epistemic resources.

The failure of application may be a result of inexperience. For example, I might know that systematically downrating women applicants constitutes discrimination, but I may not realise that this is the reason I was not called for an interview, as opposed to my CV lacking some other desirable qualification. The use of algorithms screen applications has been documented to produce similar outcomes, while their opacity limits recourse providing plausible deniability to the employers using them, as individual applicants lack the evidence to understand the reasons for their treatment. Challenging this requires opening up access to the data practices underlying the instances of algorithmic discrimination (Kelly-Lyth, 2021).

In other cases, failures of application might involve a form of gaslighting. For example, if I were offered a plausible explanation of why my application was not successful, I may accept that my lack of a certain qualification or characteristic is what caused my application to be rejected. While I may be satisfied with this explanation, a statistical analysis would show that my demographic is unfairly impacted by the policy, and that the explanation is in fact misleading. This can happen for instance if the trait that is offered in the explanation is a proxy for a protected characteristic but isn’t causally linked to the outcome of the hiring decision at hand (Wachter, 2020; Wachter et al., 2021). Even causal explanations may be problematic, as recent work has shown (Hu & Kohler-Hausman, 2020), because they rely on flawed ontological assumptions about the social categories used for classification. Cases of medical discrimination, such as the study cited above showing how African American patients had been systematically disadvantaged by a triage algorithm, often involve forms of medical gaslighting, where the algorithmically-based decisions are presented to the patient by a trusted medical professional, making it more difficult for individuals to challenge the treatment.

By contrast, social conditions that enable raising awareness and self-understanding require openness and safe venues to discuss and compare experiences. By reducing the visibility of others’ contexts, epistemic fragmentation impairs individuals’ ability to understand their own experiences through constructive comparisons, causing an inability to subsume their experience to the relevant concept. When this impairment disproportionately affects members of disadvantaged groups, who find it difficult to gain the knowledge and experience to apply the relevant concepts, fragmentation leads to a third source of hermeneutical injustice. This could be particularly insidious, since it exploits the victim’s own inability to apply the existing concept to understand their own lived experience, thus remaining hidden in plain sight.

One way in which groups might be unjustly affected by epistemic fragmentation is through a *dissonance mechanism.* To illustrate this, imagine Adam, a victim of bullying who lives in a community where negative attitudes to victims are entrenched. This could manifest, for example, in gossip or unwanted attention and judgement of the victim’s reactions which can frequently make the victim feel that they are under scrutiny. In this context, it may be difficult for Adam to come forward to others about his experiences and to correctly assess them as an instance of bullying, even if he was familiar with the concept, due to a protective psychological mechanism (Gardiner, 2021). This is despite the fact that Adam would be able to correctly assess the case under circumstances that are less charged, for example if he saw a depiction of similar behaviour in a film. A similar mechanism could be at work in cases where the harms produced by algorithmic profiling involve sensitive characteristics that carry a social stigma. Epistemically isolated victims, in these cases, may be more vulnerable to hermeneutical injustice through this defence mechanism.

A second way in which failures of application could lead to hermeneutical injustice is through repeated targeted exposure. Recent whistleblowing has revealed that Facebook conducted internal research on the relationship between the use of its social media platforms (specifically Instagram) and mental health issues, in young girls who may be at risk of developing anxiety (Heidt, 2021; Kelly et al., 2019). The users of social media in question may be aware of the power of social media to distort one’s self-perception by inviting comparison with others. For example, girls may be acutely aware that many of the pictures posted on social media are posed and use beauty filters, presenting a distorted image that does not accurately represent the reality of those who posted it. The negative effects of social media on self-perceptions are furthermore often discussed in school. Yet despite having this awareness, young girls are still negatively affected by what they see online, as shown by studies documenting increased anxiety as a result of social media exposure (Keles et al*.*, 2020). Educational campaigns may be ineffective at helping young girls to deal with the negative impacts of this messaging on the development of healthy self-perception, because they do not chime in with their emotional response, failing to take into proper account the power of repeated exposure to stereotypes. This case demonstrates how one may be in full possession of the hermeneutical resources that are needed to interpret one’s experience (e.g., of feeling physically inadequate) yet fail to take up these resources and apply them to oneself. The reason for this failure in applying the resources can be traced back to the deep entrenchment of sexist and misogynistic social norms in women’s self-understanding. These norms dictate what counts as attractive physical appearance and overemphasise their importance.

Online bullying and exposure to damaging targeted messaging illustrate well how fragmentation provides a fertile ground for hermeneutical injustice stemming from failures of application. In the fictional example from the Netflix series *Sex Education,* recalled in the opening section, Aimee’s character initially suffers from an inability to understand her own traumatic experience as an instance of sexual assault, feels disconnected and isolated as a result of her instinctive reaction of trying to brush the episode aside. This state of denial adds to the trauma, as we see Aimee’s character deal with triggering situations in ways that appear puzzling to both herself and those around her, but that start to make sense once her traumatic experience is accounted for. It is only through the involvement of other characters, who by chance learn about what happened to her and help her to open up about it, that Aimee finally starts to understand and accept the nature of her own experience. But the first steps on Aimee’s healing journey would not have been possible without the meaningful involvement of others, which only became possible when salient parts of her experience became more apparent to them, in other words when they became less epistemically fragmented. By blocking this kind of communication between peers, epistemic fragmentation can inhibit similar healing processes, thus contributing to hermeneutical injustice through failures of application.

Similar inhibitory effects on the expression and processing of individual experiences may be linked to topics that are considered socially taboo. A social taboo on mentions of menstruation, for example, can leave girls and women isolated and unable to freely express their experiences in a way that can prove to detrimental, even as the conceptual resources needed to understand them are in theory socially available, as discussed by Crear (2016).

While we have used the example of young girls on social media, our identity and self-presentation are continuously under construction and being shaped partly by our experiences online including the way in which we are profiled. Hermeneutical injustice stemming from failures of application of existing epistemic resources to our own lived experiences may be more pervasive than we realise, and our hope is that drawing attention to this mechanism will invite more research on this topic.

## Conclusion and outlook

We have argued that epistemic fragmentation, which is a characteristic feature of environments shaped by algorithmic profiling, can lead to epistemic injustice by sustaining the infrastructural conditions where new epistemic resources are i) difficult to produce; ii) fail to be up taken by the relevant decision makers and reflected in the policies to regulate the practice of algorithmic profiling; and finally iii) it remains difficult for individuals to learn to apply the existing epistemic resources to their own lived experiences. By now, it should be clear that therefore epistemic fragmentation should be seen as an infrastructural flaw of epistemic environments, contributing to *infrastructurally produced* hermeneutical injustice.

The infrastructural conditions of fragmentation disproportionately affect marginalised and disadvantaged groups, and therefore are a source of epistemic injustice. Fixing the other sources of hermeneutical injustice requires overcoming the structural issues brought about by fragmentation.

From a bottom-up perspective, maintaining epistemic fragmentation leads to individuals being less equipped to understand and communicate their algorithmically mediated experiences, leaving them vulnerable to abuse. This normalising of individual harms, hiding of hermeneutical resources, depleting motivation and resources to uncover discrimination, undermines the ability and motivation of individuals to care for others, for example through the creation of support networks. Harmful and wrongful interactions that are mediated by algorithmic profiling are less likely to be identified in the absence of external signals that could alert them of something being amiss. Discrimination is harder to detect and prove. In order to identify discrimination, one needs access to population-level information, together with the motivation to investigate the appropriate patterns and characteristics on which they are predicated, all of which is obscured in epistemically fragmented environments.

From a top-down perspective, too, the governance structures for algorithmic profiling systems are not sufficiently accountable. Oversight bodies are not able to produce new hermeneutical resources, draw on those that are already present or offer meaningful opportunities for the affected communities to participate in the monitoring of the system’s behaviour. The trend to resort to regulation and increased proactive monitoring of algorithmic profiling has significant cost and is unlikely to be successful (Milano et al., 2021). Moreover, it will lead to the worsening of lacunas if the regulators do not attempt to ameliorate the production, uptake and application of epistemic resources involving the affected groups.

In this paper, we have considered the ways in which dysfunctions of epistemic infrastructure can lead to epistemic injustice and proposed a framework to conceptualise them, with the aim to provide tools which will help to identify, and potentially address, issues that might be arising due to epistemic fragmentation. For example, understanding the risks that epistemic fragmentation poses in the context of personalised social media helps to identify ways in which the issues might be addressed, for instance reducing the extent of personalised targeting, creating searchable repositories for social media content shown to different audience segments to permit auditing, and find alternative mechanisms to allow users of social media to share and compare their experiences, by altering the platform design. While a treatment of the possible solutions to the issues of epistemic injustices raised by algorithmic profiling lies outside the scope of this paper, our discussion shows that any such solutions will need to target the epistemic infrastructure, to enable community-building and sharing of epistemic resources. It will not be enough to think only of how the infrastructure impacts one element, for instance concept formation. Instead, addressing the sources of hermeneutical injustice that we have identified here will require a holistic approach (Greene et al., 2023). It might be easy to share information on social media, but if the infrastructure does not support meaningful opportunities to uptake and assimilate valuable new epistemic resources, then this will not create the conditions for epistemic justice. The strategy to address the negative consequences of epistemic fragmentation will vary depending on the context and systems concerned (Gillespie et al., 2020, Kirk et al., 2023). Initial steps should include increasing transparency and researchers’ access to data about targeting behaviour that is necessary to permit oversight by civil society (Greene et al., 2022). In some instances, targeting might need to be banned altogether, limited to specific lower-risk contexts, or audited using algorithmic tools to monitor the extent to which it leads to fragmentation among individuals (Laux et al., 2022), protect their privacy (Véliz, 2021) and enable more democratic models of oversight for algorithmic profiling systems.

## Data Availability

The authors declare that no new data were created or analysed during this research. Data sharing is not applicable to this article.
